# Introgression from cultivated rice alters genetic structures of wild relative populations: implications for *in situ* conservation

**DOI:** 10.1093/aobpla/plx055

**Published:** 2017-10-16

**Authors:** Xin Jin, Yu Chen, Ping Liu, Chen Li, Xingxing Cai, Jun Rong, Bao-Rong Lu

**Affiliations:** Ministry of Education, Key Laboratory for Biodiversity Science and Ecological Engineering, Department of Ecology and Evolutionary Biology, Fudan University, Shanghai, China; Rice Research Institute, Guangdong Academy of Agriculture Sciences, Guangzhou, Guangdong Province, China; Center for Watershed Ecology, Institute of Life Science and Key Laboratory of Poyang Lake Environment and Resource Utilization, Ministry of Education, Nanchang University, Nanchang, China

**Keywords:** Crop wild relatives, gene flow, genetic relationship, molecular genotyping, natural hybridization, *Oryza rufipogon*

## Abstract

Maintaining genetic integrity is essential for *in situ* and *ex situ* conservation of crop wild relative (CWR) species. However, introgression of crop alleles into CWR species/populations may change their genetic structure and diversity, resulting in more invasive weeds or, in contrast, the extinction of endangered populations. To determine crop-wild introgression and its consequences, we examined the genetic structure and diversity of six wild rice (*Oryza rufipogon*) populations under *in situ* conservation in China. Thirty-four simple sequence repeat (SSR) and 34 insertion/deletion markers were used to genotype the wild rice populations and two sets of rice cultivars (*O. sativa*), corresponding to the two types of molecular markers. Shared alleles and STRUCTURE analyses suggested a variable level of crop-wild introgression and admixture. Principal coordinates and cluster analyses indicated differentiation of wild rice populations, which was associated with the spatial distances to cultivated rice fields. The level of overall genetic diversity was comparable between wild rice populations and rice cultivars, but a great number of wild-specific alleles was detected in the wild populations. We conclude based on the results that crop-wild introgression can considerably alter the pattern of genetic structure and relationships of CWR populations. Appropriate measures should be taken for effective *in situ* conservation of CWR species under the scenario of crop-wild introgression.

## Teaser

We found gene introgression from cultivated rice to its in situ conserved wild relative populations, which has considerably altered their genetic structure and relationship. The level of introgression is associated with the spatial distances of the wild populations from cultivated rice fields. The findings are based on genetic analyses of six wild populations and two sets of rice cultivars using two types of molecular markers. Crop-wild introgression alters the genetic integrity of wild populations. Thus, appropriate measures should be taken for effective in situ conservation of wild relatives of crop cultivars.

## Introduction

Sustaining genetic diversity of crop wild relative (CWR) species is essential for crop genetic improvement (Ellstrand and Elam 1993), although it is challenging to maintain genetic integrity of CWR species/populations in the changing environment. Introgression (incorporation of alleles from one differentiated population to another) of crop alleles to CWR species may significantly change their genetic diversity (Ellstrand *et al.* 2013). Introgressed alleles conveying beneficial or detrimental traits may result in considerable genetic changes in the recipient populations (Baack and Rieseberg 2007), leading to the evolution of more aggressive invasive species or, in contrast, the extinction of endangered species under natural selection (Ellstrand 2003). For instance, introgression of cultivated carrots (*Daucus carota* ssp. *sativus*) with their wild relatives (*D. carota* ssp. *carota*) resulted in adjacent wild relative populations genetically similar to cultivars, which stimulated the evolution of more aggressive weeds in carrot fields (Magnussen and Hauser 2007). The opposite examples of crop-wild introgression can be found in cultivated (*Raphanus sativus*) to wild radishes (*R. raphanistrum*) in California, which caused the extinction of local wild radishes (Hegde *et al.* 2006). Likewise, introgression from cultivated yams (*Dioscorea rotundata*) to wild yams (*D. abyssinica* and *D. praehensilis*) might be the reason for the local extinction of wild yams in West Africa (Scarcelli *et al.* 2017).

As demonstrated by Todesco *et al.* (2016), introgression may drive endangered species to extinction through demographic or genetic swamping. In the case of demographic swamping (introgression-resulted hybrids are unfit and entirely removed), growth rate of endangered species is reduced due to the wasteful production of maladaptive hybrids. For genetic swamping (introgression-resulted hybrids are partially fertile/viable and replace pure parental genotypes), genetically pure parental genotypes can get lost from endangered species by the replacement of the introgression-resulted hybrids (see Fig. 1 in Todesco *et al.* 2016 for details). Extinction caused by genetic swamping of crop-wild introgression might be more frequent than extinction caused by demographic swamping in agroecosystems (Todesco *et al.* 2016). Therefore, identifying the types of introgression (demographic or genetic swamping) may help to predict the risk of extinction for endangered CWR species.

Recently, introgression of selectively advantageous transgenes from genetically engineered (GE) crops to their CWR populations through pollen-mediated gene flow has aroused worldwide biosafety concerns because of the potential environmental impact and increased difficulties for CWR *in situ* conservation (Andow and Zwahlen 2006; Lu and Yang 2009; Lu 2016). Increased seed production in wild sunflower (*Helianthus annuus*) caused by introgression of an insect-resistant transgene (*Bt*) from cultivated sunflower exemplified the potential of enhanced invasiveness in wild sunflowers (Snow *et al.* 2003). Enhanced fecundity reported in weedy rice (*Oryza sativa* f. *spontanea*) by introgression of a glyphosate-resistant transgene from crop rice (Wang *et al.* 2014) may also cause potential environmental problems. Thus, improved understanding of crop-wild introgression can facilitate the design of effective CWR conservation strategies and accumulate baseline data for assessing potential environmental impact of transgene introgression (Lu and Yang 2009; Lu 2013, 2014).

Perennial common wild rice (*Oryza rufipogon*; referred to as W hereafter) is the wild ancestor of cultivated rice (*O. sativa*) and is extensively distributed in the tropics and subtropics of Asia (Oka 1988). W is valuable germplasm for the genetic improvement of cultivated rice and for studying rice domestication and evolution. China is the northernmost distribution range of W found in southern provinces (Song *et al.* 2003b). However, W has become endangered in China in the past few decades, due to substantial changes in farming systems, land uses, urbanization and other human activities (Song *et al.* 2003b; Lu 2013). Consequently, *in situ* conservation for this species was initiated in China (Wang *et al.* 2009). However, it is recognized that crop-wild introgression may challenge the current conservation methods, in which the W populations are only wire-fenced from their nearby cultivars. Studies of crop-wild gene flow and introgression are reported in rice (Song et al. 2003b, 2006), but little is known about the impact of such introgression on genetic structures and diversity of W populations.

Molecular markers are powerful tools to identify crop-wild introgression (Song *et al.* 2003a; Chen *et al.* 2004; Ellstrand *et al.* 2013). Simple sequence repeats (SSRs) or microsatellites, containing tandem repeats of short sequences (2–6 nucleotides), can be used as a powerful tool to identify genetic variation at a given locus in population studies. The insertion/deletion (InDel) markers based on the BLAST (basic local alignment search tool) of DNA sequences between the total genomes of a typical *indica* rice variety (93-11) and *japonica* rice variety (Nipponbare) were originally developed to identify *indica* and *japonica* traits of rice (Lu *et al.* 2009). Further studies showed that InDel markers were particularly useful to determine genetic relationships of cultivated rice and wild/weedy rice populations (Xiong *et al.* 2011; Liu *et al.* 2012), in addition to genetic diversity and structures (Song *et al.* 2015).

We studied potential crop-wild introgression involving six W populations with different spatial distances to rice fields, using SSR and InDel markers. We included two sets of rice cultivars collected from southern China (for SSR analysis) and Gaozhou (for InDel analysis) as separate references. We attempt to address the following questions in this study. (i) Does introgression occur from cultivated rice to its wild progenitor? (ii) Does crop-wild introgression affect genetic relationships and structure of the recipient W populations? (iii) Does crop-wild introgression alter genetic diversity of the recipient W populations? The generated knowledge will be useful for understanding the role of crop-wild introgression in shaping the genetic structure and diversity of CWR populations, and for designing strategies for effective *in situ* conservation of CWR species, particularly in the close vicinity of their cultigens.

## Methods

### Study site

**Figure 1.**
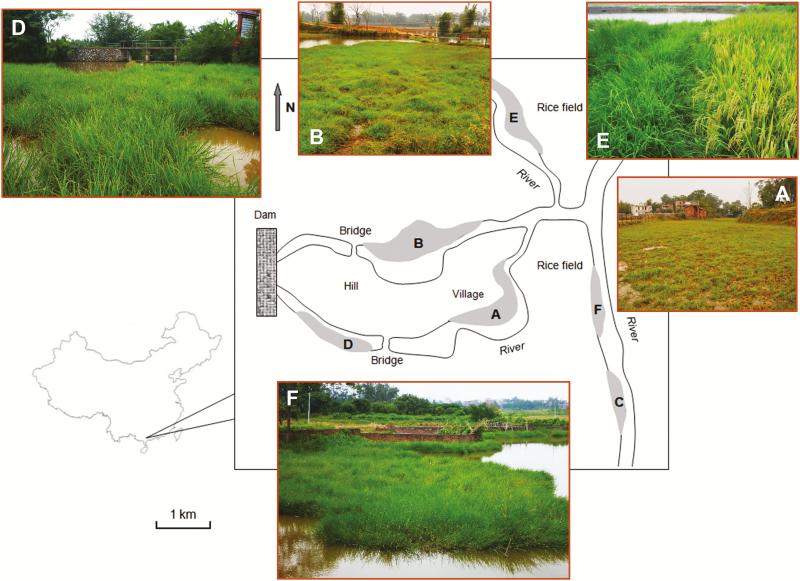
The locations and habitats of wild rice (*Oryza rufipogon*) populations in Gaozhou, Guangdong Province, China. A–F indicate the six *O. rufipogon* populations that are highlighted by shady areas. Population-A (photo A), -B (photo B), -C and -D (photo D) located at canals relatively far away (~100 m) from rice fields. Population-E (photo E, wild rice on the left and cultivated rice on the right) and -F (photo F) located along a canal near (5–10 m) rice fields.

Six natural W populations scattered in an area of ~30 km^2^ in Gaozhou of Guangdong Province in China were included for this study (Fig. 1), where the estimated rice cultivated areas are >100 ha in the neighbourhood. These W populations are currently under the national *in situ* conservation programme because this area is an important centre of *O. rufipogon* diversity in China. The W populations occurred along rivers across different villages under slightly different habitats (Table 1; Fig. 1). The A-, B- and D-population occurred along the river where a concrete dam (photo D in Fig. 1) was built to accumulate water for irrigation. Therefore, the three W populations were under a deep-water condition with a more or less homogenous habitat. The C-population was almost isolated (~100 m) from all other W populations in shallow water. Nearly no cultivated rice was found in the vicinity (~50 m) of the four W populations (A, B, C and D, Fig. 1), except for the A-population where farmers sporadically planted rice. The E- and F-population occurred on shallow-water riverbanks, with different rice cultivars nearby (5–10 m), particularly for the E-population (photos E and F in Fig. 1).

**Table 1.** Populations of perennial common wild rice (*Oryza rufipogon*) and cultivated rice (*O. sativa*) included in this study with information on sample size and location/origin in China.

Wild/cultivated rice	Code	No. of individuals/cultivars	Location and origin (village, district, county, province)
Population-A	A	50	Fushi, Zhenjiang, Gaozhou, Guangdong
Population-B	B	44	Pengshan, Zhenjiang, Gaozhou, Guangdong
Population-C	C	38	Daling, Zhenjiang, Gaozhou, Guangdong
Population-D	D	10	Boshui, Zhenjiang, Gaozhou, Guangdong
Population-E	E	24	Shatian, Zhenjiang, Gaozhou, Guangdong
Population-F	F	45	Tanlu, Zhenjiang, Gaozhou, Guangdong
Cultivars from gene banks of southern China	CV-1	35	Guangdong Province, Guangxi Province, Fujian Province
Cultivars from Gaozhou	CV-2	50	Gaozhou and its neighbouring regions in Guangdong Province

### Materials

Leaf samples were collected from different individuals of the six W populations (10–50 individuals) and placed separately in zip-lock plastic bags containing silica gels for dehydration. The dry leaf samples were stored in a freezer at −20 °C until used. Two independent sets of rice cultivars (CV) were included in the analyses. The first set (coded as CV-1) contained 35 cultivars collected from southern China, including Guangdong, Guangxi and Fujian provinces (Table 1; **see**Supporting Information—Table S1). The second set (coded as CV-2) contained 50 cultivars from Gaozhou and its neighbouring area (Table 1; **see Supporting Information—Table S2**). Seed samples of all rice cultivars were donated by the Guangdong Academy of Agriculture Sciences (Guangzhou) and the Chinese National Gene Bank (Beijing).

### DNA extraction and PCR assay

The total genomic DNA was extracted from leaf samples using the modified CTAB protocol described by Song *et al.* (2003b). DNA was extracted from dry leaves for W and fresh leaves for CV from three-leaf-stage seedlings.

Thirty-four SSR primer pairs from the rice genome (http://www.gramene.org) and 34 InDel primer pairs developed by Lu *et al.* (2009) were selected to analyse genetic parameters of Gaozhou W populations and the two sets of rice cultivars. The SSR and InDel loci were distributed across the 12 rice chromosomes **[see Supporting Information—Tables S3 and S4]**. PCR reaction was performed in ABI (Applied Biosystems Inc., USA) Thermocycler using the following programme: a denaturation period of 4 min at 94 °C followed by 28 cycles of 30 s at 94 °C, 30 s at 55 °C and 40 s at 72 °C, and then 7 min at 72 °C for final extension. Reactions were carried out in a volume of 10 μL containing 1× buffer, 2.5 mM each of dATP, dCTP, dGTP and dTTP, 10 μM of SSR or InDel primer, 50 ng of genomic DNA and 0.6 unit of *Taq* polymerase (TaKaRa Inc.). PCR products were separated in 4 % denaturing polyacrylamide gel (PAGE). The electrophoretic bands were revealed using a silver staining procedure. The gel was washed twice with ddH_2_O and then placed into a 200–400 mL solution of 0.1 % AgNO_3_ solution to stain for 10–15 min. The stained gel was washed twice by 200–400 mL ddH_2_O, and then placed into a 1.5 % NaOH solution (about 200–400 mL) containing 0.4 % formaldehyde for about 10 min to obtain visible DNA bands. Photographs were taken from the PAGE gels using a digital camera (Sony Inc.).

### Data scores and analysis

The co-dominant SSR electrophoretic banding patterns were scored as either a homozygous or heterozygous genotype, based on their molecular weight estimated by the pUC19 DNA/MspI (HpaII) marker ladder (Fermentas International Inc.). The dimorphic InDel markers were also co-dominant and their electrophoretic banding patterns were scored as a homozygous AA or BB genotype, or a heterozygous AB genotype following the standard of Lu *et al.* (2009) and Liu *et al.* (2012).

The proportions of pairwise shared alleles (ps = Σ_*k*_Σ_*a*_min(*f*_*a*,*i*_, *f*_*a*,*j*_)/*D*) among W populations and CVs either at the 34 SSR loci (CV-1) or 34 InDel loci (CV-2) were calculated, respectively, using the software Microsatellite Analyser (MSA) version 4.00 (Dieringer and Schlötterer 2003), where *f*_*a*,*i*_/_*a*,*j*_ indicates the frequency of the allele *a* at the locus *k* in the population *i*/*j*, and *D* represents the number of loci. In addition, the frequencies of shared alleles by rice cultivars and W populations at different SSR and InDel loci were also calculated to assess possible introgression using the software GenAlEx ver. 6.5 (Peakall and Smouse 2012).

The software STRUCTURE version 2.3.4 was used to determine the population genetic structure and admixture (Pritchard *et al.* 2000) among W populations and cultivars based on the SSR and InDel data matrices, respectively. The admixture model and correlated allele frequencies model were applied. Population number (*K*) was evaluated from 1 to 8 and 10 replicates were run for each *K*. Each run had a burn-in period of 100000 and 200000 iterations after burn-in. The software CLUMPP ver. 1.1.2 was used to find the optimal alignments of the 10 replicates with the ‘FullSearch’ algorithm (Jakobsson and Rosenberg 2007). The clustering results were visualized using the software Distruct ver. 1.1 (https://web.stanford.edu/group/rosenberglab/distructDownload.html). The highest log-likelihood value indicated the closest test to the actual number of genetically distinct populations. If an individual’s genotype profile indicated admixture, it would be assigned to a population with the same colour code. The W populations that were introgressed with the cultivar population would then be determined.

Data matrices generated based on the 34 SSR and InDel primer pairs were subjected to the principal coordinates analysis (PCoA) to estimate genetic relationships and structure among W populations, using rice cultivars as a reference. The scatter plot was generated based on the values of correlation coefficient of the first two principal coordinates. A UPGMA dendrogram was constructed based on the pairwise genetic distance (Nei 1978) to estimate genetic relationships of W populations and CVs. The Mantel test was performed (1000 iterations) to examine the correlation between genetic and spatial distances among W populations at different localities. The pairwise *F*_st_ was calculated to estimate the level of genetic differentiation among W populations and CVs. Genetic diversity parameters of W populations and CV-1 rice cultivars were estimated based on the SSR markers. All above analyses were performed using the software GenAlEx ver. 6.5 (Peakall and Smouse 2012).

## Results

### Possible allelic introgression from cultivated rice

The proportion of shared alleles by the two sets of rice cultivars (CV-1 and CV-2) with the corresponding W populations at the 34 SSR and InDel loci was calculated, compared with the shared alleles among W populations. In general, a greater proportion of pairwise shared alleles was detected among W populations than that between the W populations and the two sets of rice cultivars (Table 2), likely due to their outbreeding features. Still, considerable frequencies of shared alleles were detected at 16 of the 34 analysed SSR loci between W populations and the two sets of rice cultivars although no shared wild-crop alleles were found at other loci **[see Supporting Information—Table S5]**. However, the proportion of shared alleles of the two sets of rice cultivars with their corresponding W populations varied considerably. Noticeably, the E-population nearby rice fields showed the highest proportion of shared alleles with the two sets of rice cultivars, with ~41 % with CV-1 (SSR markers) and ~64 % with CV-2 (InDel markers), respectively. On the other hand, the C-population far from rice fields showed the lowest proportion of shared alleles with CV-1 (~18 %, SSR markers), although the proportion of shared alleles with CV-2 (~45 %, InDel markers) was similar to other wild populations (Table 2).

**Table 2.** Proportion of shared alleles (ps = Σ_*k*_Σ_*a*_min(*f*_*a*,*i*_, *f*_*a*,*j*_)/*D*) between wild rice (*Oryza rufipogon*) populations and the two sets of rice cultivars (*O. sativa*) at the 34 SSR loci (below diagonal) and 34 InDel loci (above diagonal). In the formula, *f*_*a*,*i*_/_*a*,*j*_ is the frequency of the allele *a* at the locus *k* in the population *i*/*j*; *D* is the number of loci. Bold numbers show the highest values in the two sets of rice cultivars.

Population code	A	B	C	D	E	F	CV-2
A	–	0.802	0.794	0.788	0.657	0.863	0.389
B	0.501	–	0.750	0.840	0.730	0.876	0.440
C	0.361	0.377	–	0.818	0.745	0.805	0.451
D	0.600	0.497	0.393	–	0.748	0.829	0.464
E	0.415	0.382	0.349	0.425	–	0.691	**0.644**
F	0.475	0.467	0.418	0.517	0.406	–	0.415
CV-1	0.276	0.339	0.176	0.336	**0.409**	0.260	–

To gain more detailed information about the shared alleles between the Gaozhou W populations and rice cultivars from southern China (CV-2), we analysed the frequency of abundant SSR alleles (>15 %) at various loci of the rice cultivars in each of the W populations. Our results showed that many of the SSR alleles detected in the rice cultivars with high frequencies (up to 91 %) were also present in the W populations, although a large number of W-specific alleles (105) and cultivar-specific alleles (61) were detected. Apparently, these SSR alleles detected in the rice cultivars (CV-2) with high frequencies were present in W with considerable variation among populations **[see Supporting Information—Table S5]**. Interestingly, the E-population showed a relatively high frequency of alleles that were abundant in rice cultivars (CV-2) at a few SSR loci, indicating possible introgression of cultivar alleles to this W population. On the other hand, all the cultivar abundant alleles were not detected in the C-population, suggesting no or extremely limited introgression of cultivar alleles to this population. In addition, most of the InDel alleles were shared by the rice cultivars (CV-2) and W populations, without a clear pattern **[see Supporting Information—Table S6]**. This result suggested that the dimorphic InDel markers had limited uses for detecting introgression in rice.

**Figure 2.**
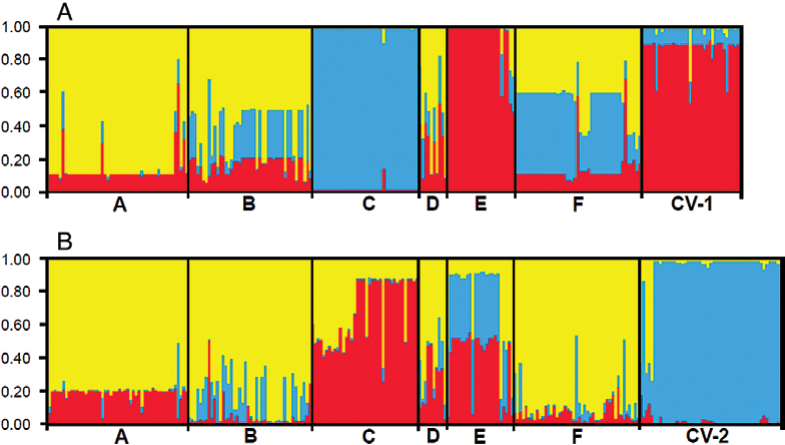
The model-based clusters of wild *Oryza rufipogon* individuals from six populations (A to F) and two sets of rice cultivars from Guangdong, Guangxi and Fujian provinces (CV-1) and Gaozhou (CV-2) based on the STRUCTURE analysis (*K* = 3) of SSR markers (panel A) and InDel markers (panel B). Identities of the wild populations and the cultivar groups are indicated below the figures (vertical bars) divided by black lines. The height of different colours in each individual designates its membership coefficient to the different clusters, and the same colour indicates the close consanguinity.

Results from STRUCTURE analyses indicated relatively similar admixture patterns of the six W populations and the corresponding cultivars, based on the SSR (*K* = 3) and InDel (*K* = 3) data matrices (Fig. 2A and B). Many individuals in the W populations exhibited a multiple cluster assignments (yellow, blue and red) based on both SSR and InDel markers, implying an admixed genetic components for most of these individuals. However, different W populations showed their unique identity (Fig. 2A and B). The two sets of rice cultivars (CV-1 and CV-2) were assigned into unique groups, respectively, from most of the W populations. Interestingly, the E-population shared a high proportion of cultivar components from the two sets of molecular markers, and in contrast, the C-population showed a different genetic composition from the other W populations and little consanguinity with cultivars (Fig. 2A and B). These results generally indicated possible crop-wild introgression.

### Genetic structure and differentiation of wild rice populations

**Figure 3.**
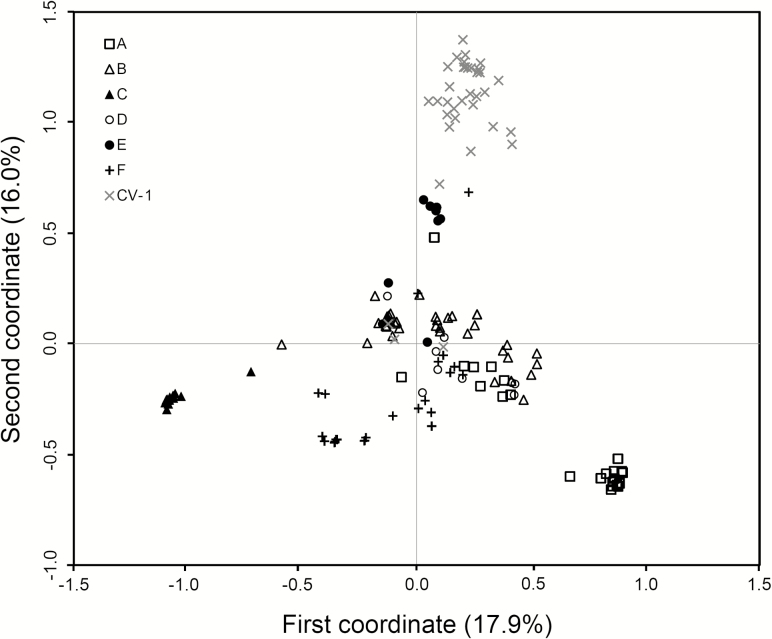
The scatter plot showing genetic relationships of 221 wild rice (*Oryza rufipogon*) individuals in six populations (A to F, indicated by different symbols in the figure) and a set of rice cultivars (CV-1) from Gaozhou of southern China generated from the coefficients of the first two principal coordinates based on PCoA of the SSR-marker data matrix. In the plot, the majority of rice cultivars formed an independent group with a few cultivars scattered within the wild rice populations. Some samples in the E-population are relatively closely associated with rice cultivars.

**Figure 4.**
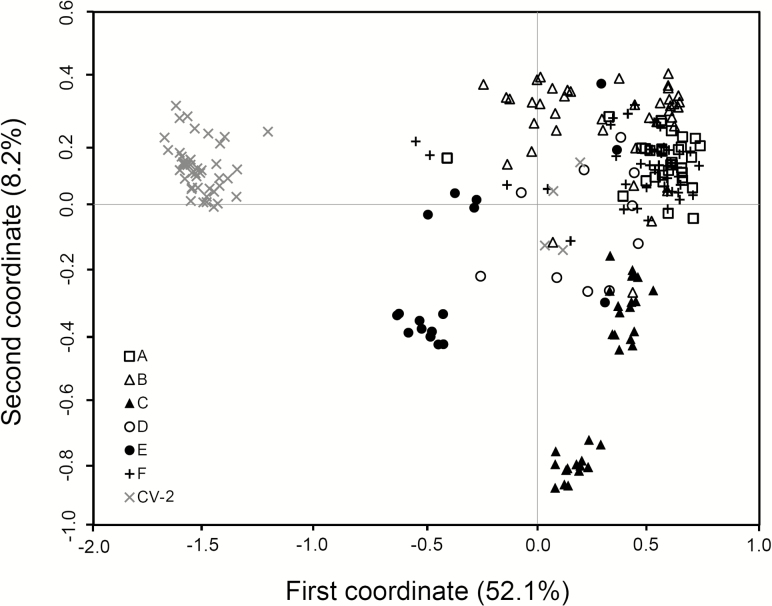
The scatter plot showing genetic relationships of 221 wild rice (*Oryza rufipogon*) individuals in six populations (A to F, indicated by different symbols in the figure) and a set of rice cultivars (CV-2) from Gaozhou of southern China generated from the coefficients of the first two principal coordinates based on PCoA of the InDel-marker data matrix. In the plot, the majority of rice cultivars formed an independent group with a few cultivars scattered within wild rice populations. Some samples in the E-population are relatively closely associated with rice cultivars.

In general, W populations from Gaozhou with the reference of rice cultivars showed considerable genetic differentiation, although these populations were scattered in a relatively small region within about 30 km^2^. The estimation of genetic relationships of the W populations was made based on the PCoA, using both SSR and InDel fingerprints, respectively (Figs 3 and 4). For SSR data matrices developed from 221 W samples in the six W populations, the scatter plot of PCA showed a clear pattern of a three-group classification (Fig. 3). All samples in the A-, B-, D- and F-population were clustered in one group, and nearly all samples in the C- and E-population were distributed in two distinctly separated groups along the first two principal coordinates, explaining 33.9 % of total variation (Fig. 3). In addition, some individuals from E-population were close to rice cultivars suggesting introgression from cultivars (Fig. 3). For the InDel data matrices including 221 W samples, the scatter plot did not show a very clear three-group pattern along the first two principal coordinates that accounted for 60.3 % of total variation. However, the separation of the C- and E-population from other W populations was apparent (Fig. 4). Notably, the C- and E-population exhibited a comparatively distinct genetic relationship from all other populations, based on both SSR and InDel fingerprints (Figs 3 and 4). Altogether, these results indicated that W populations had considerable genetic differentiation, particularly, for the C- and E-population that were relatively distinct from all other populations. It is therefore necessary to identify what are the potential reasons for the differentiation of the W populations under the relatively homogenous environment.

**Figure 5.**
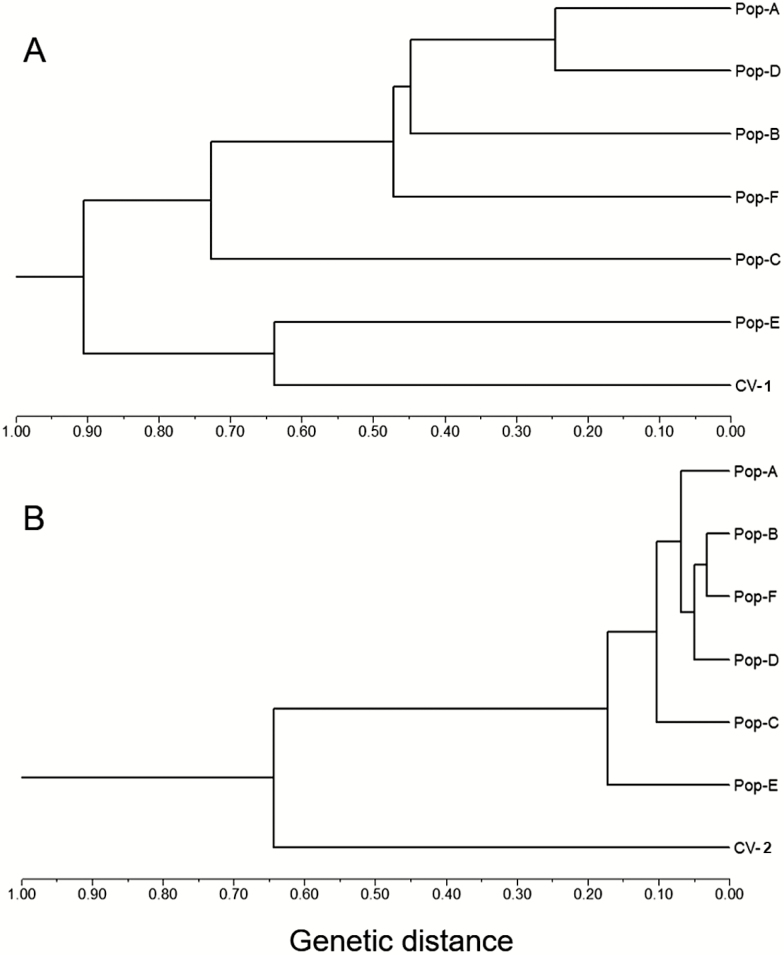
UPGMA dendrogram showing genetic relationships of six wild *Oryza rufipogon* populations (Pop-A to Pop-F) and the two corresponding sets of rice cultivars (CV-1 for SSR and CV-2 for InDel) based on the Nei (1978) genetic distance calculated from the SSR (panel A) and InDel (panel B) data matrices, respectively. The dendrogram based on both SSR and InDel markers indicates relatively close genetic relationships of cultivated rice with the E-population.

A UPGMA dendrogram was constructed based on pairwise genetic distances (Nei 1978) to estimate genetic relationships of W populations, using rice cultivars (CV-1 and CV-2) as references. The dendrogram based on SSR data matrix indicated two major groups at the distance coefficient of 0.91, with five W populations (A, B, C, D and F) in one group and CV-1 and the E-population in another group (Fig. 5A). In the first group, the C-population was separated from other populations. The results suggested a relatively close relationship of rice cultivars with the E-population, and a relatively distant relationship of the C-population with all other populations. The dendrogram based on InDel data matrix also indicated two major groups at the distance coefficient of 0.64, with the CV-2 group quite separated from all six W populations (A–F) that were clustered in one group (Fig. 5B). In the W group, the E- and C-population were quite separated from other W populations.

Pairwise *F*_st_ was calculated to estimate the level of genetic differentiation among the W populations and rice cultivars (CV-1 and CV-2) (Table 3). The level of genetic differentiation was low to moderate (*F*_st_ = 0.13–0.37 for SSR; *F*_st_ = 0.04–0.22 for InDel) among W populations. Noticeably, the lowest values of genetic differentiation were observed between the E-population and the rice cultivars based on both SSR and InDel data matrices (Table 3), suggesting considerable crop-wild introgression. The mantel test based on the SSR data matrix did not show significant correlation (*R*^2^ = 0.012, *P* = 0.38 for genetic distance; *R*^2^ = 0.001, *P* = 0.40 for *F*_st_) of the pairwise genetic distance or genetic differentiation (*F*_st_) with the geographic distance of the W populations, suggesting no isolation-by-distance pattern in terms of genetic divergence of the W populations.

**Table 3.** Genetic differentiation (pairwise *F*_st_) between wild rice (*Oryza rufipogon*) populations and the two sets of rice cultivars (*O. sativa*, CV-1 and CV-2) at the 34 SSR loci (below diagonal) and 34 InDel loci (above diagonal). Bold numbers indicate the lowest values of genetic differentiation between wild rice and the corresponding rice cultivars.

Population code	A	B	C	D	E	F	CV-2
A	–	0.098	0.125	0.108	0.220	0.065	0.502
B	0.189	–	0.137	0.063	0.129	0.043	0.394
C	0.367	0.250	–	0.089	0.136	0.100	0.420
D	0.130	0.130	0.247	–	0.122	0.068	0.368
E	0.261	0.189	0.318	0.175	–	0.161	**0.203**
F	0.215	0.168	0.256	0.137	0.225	–	0.442
CV-1	0.270	0.187	0.322	0.175	**0.167**	0.249	–

### Genetic diversity of *O. rufipogon* populations

We included data matrices from SSR and InDel markers to estimate genetic diversity of the W populations using the two sets of rice cultivars (CV-1 and CV-2) as references. All the 34 selected SSR loci were polymorphic in W populations and rice cultivars. A total of 326 alleles were recorded in all samples with the highest number of alleles per locus at RM228 (18 alleles) and the lowest at RM29, RM130, RM16, RM348, RM598 and RM245 (five alleles). Results of other diversity parameters, such as the number of alleles (*A*) and effective alleles (*A*_e_), observed heterozygosity (*H*_o_), expected heterozygosity (*H*_e_), Shannon Index (*I*) and Nei’s unbiased genetic diversity (Nei 1978), were listed in Table 4. As indicated by the Nei’s diversity parameter (Nei 1978), the overall genetic diversity of W and rice cultivars were comparable, although considerable variation was detected among W populations (ranging from ~0.32 to 0.63). Interestingly, the observed heterozygosity (*H*_o_ = 0.81) of the E-population was considerably greater than that of all other W populations, although the level of its genetic diversity was comparable with the other W populations. On the other hand, genetic diversity of the C-population showed the lowest value in nearly all the parameters, except for the observed heterozygosity. Noticeably, a much greater number of wild-specific alleles was detected in the W populations than that of crop- specific alleles in rice cultivars (Table 4), suggesting abundant and unique genetic variation in the W populations. A different pattern regarding genetic diversity of the W populations and cultivated rice was observed based on the dimorphic InDel markers, except for the observed heterozygosity (*H*_o_) that show a similar trend with the highest value in the E-population **[see Supporting Information—Table S7]**. In addition, no specific alleles to rice cultivars or wild rice populations were detected **[see Supporting Information—Table S7]**.

**Table 4.** Genetic diversity parameters for wild rice (*Oryza rufipogon*) populations (A–F) in Gaozhou compared with those of cultivated rice controls (CV-2 and CV-1) from southern China based on SSR fingerprints. ^1^*A*: number of alleles; *A*_e_: number of effective alleles; *H*_o_: observed heterozygosity; *H*_e_: expected heterozygosity; *I*: Shannon index; DG: Nei’s unbiased genetic diversity (Nei 1978). Numbers in parentheses indicate SD. ^2^The overall values of genetic diversity parameters were calculated based on all wild rice samples as one population. (The bold value indicates the highest observed heterozygosity among populations.)

Population code	*A* ^1^	*A*_e_	*H*_o_	*H*_e_	*I*	DG	Total no. of alleles	No. of alleles specific to rice cultivars or wild rice populations
Wild rice-A	4.059 (1.301)	1.662 (0.553)	0.450 (0.441)	0.335 (0.199)	0.609 (0.297)	0.339 (0.034)	138	15
Wild rice-B	4.088 (1.525)	2.603 (1.109)	0.591 (0.341)	0.555 (0.173)	1.001 (0.384)	0.562 (0.030)	139	8
Wild rice-C	2.118 (0.844)	1.639 (0.533)	0.581 (0.488)	0.311 (0.25)	0.462 (0.361)	0.315 (0.043)	72	4
Wild rice-D	3.265 (1.287)	2.600 (0.88)	0.641 (0.363)	0.569 (0.159)	0.981 (0.358)	0.600 (0.029)	110	4
Wild rice-E	3.412 (1.104)	2.051 (0.434)	**0.806** (0.349)	0.483 (0.148)	0.791 (0.238)	0.493 (0.026)	116	8
Wild rice-F	4.235 (1.827)	2.111 (0.781)	0.502 (0.427)	0.450 (0.23)	0.818 (0.433)	0.455 (0.040)	142	11
Overall^2^	7.471 (2.631)	3.217 (1.268)	0.564 (0.261)	0.648 (0.124)	1.323 (0.345)	0.461 (0.016)	254	108
CV-1	6.353 (2.973)	3.473 (1.936)	0.134 (0.308)	0.605 (0.232)	1.292 (0.590)	0.623 (0.040)	215	71

## Discussion

Our results based on SSR and InDel molecular markers indicated a considerable proportion of shared alleles among the Gaozhou W populations, and between the W populations and the two corresponding sets of rice cultivars. STRUCTURE analyses of the SSR and InDel data matrices also indicated the admixture between the W populations and rice cultivars. These results have addressed our first question about crop-wild allelic introgression at the wild rice *in situ* conservation site in Gaozhou. Therefore, we consider that the level of introgression is much higher among the W populations than that between the W populations and rice cultivars, probably attributing to the considerably higher outcrossing rates of the wild *O. rufipogon* (Oka 1988). In addition, the extent of crop-wild introgression appears to be associated with the spatial distances between the W populations and cultivated rice fields, which can be exemplified by the results of crop-wild introgression of the E- and C-population.

Previous studies showed that the flowering period of *O. rufipogon* overlapped with that of cultivated rice (Song *et al.* 2002, 2003a). A considerable frequency (~3–18 %) of pollen-mediated gene flow, negatively correlating with the increased spatial distances from pollen donors (cultivar), was detected from cultivated rice to *O. rufipogon* under the field experimental conditions (Song *et al.* 2003a; Wang *et al.* 2006), supporting the results of our observed rice crop-wild introgression. These findings are in line with the previous study where introgression of cultivated carrots to wild carrots resulted in wild carrot populations adjacent to cultivated carrot fields genetically more similar to cultivated carrots than wild carrot populations far from cultivation fields (Magnussen and Hauser 2007). Therefore, the level of crop-wild introgression is associated with spatial distances between crops and their CWR populations/species.

Results from PCoA and cluster analyses of SSR and InDel molecular markers indicate considerable influences of crop-wild introgression on genetic relationships of W populations with cultivars in a given region. For example, the E-population that was spatially close to rice fields with relatively frequent crop-wild introgression showed a relatively close relationship with rice cultivars, whereas the C-population that was spatially distant from rice fields (also from other W populations) showed a relatively distant relationship with rice cultivars based on PCoA data. A similar pattern of genetic relationships between W populations and cultivars was also found based on UPGMA cluster analyses. Notably, the E-population was clustered together with rice cultivars in the dendrogram constructed based on SSR data matrix, although no significant correlation between the genetic and spatial distances was detected based on the Mantel test. In addition, the pairwise *F*_st_ values suggested considerable genetic differentiation among the W populations, with the lowest level of differentiation between the E-population and rice cultivars. All these results have answered our second question about the impact of crop-wild introgression on the genetic relationship and structure of the target W populations.

Our results did not show considerably high genetic diversity of the E-population, which has answered our third question raised earlier about genetic diversity of W populations. That is crop-wild introgression did not alter overall genetic diversity of the W populations. However, the studied W populations contained a much greater number of wild-specific alleles, suggesting relatively abundant and unique genetic variation in the W gene pool in Gaozhou. The unique genetic variation in wild rice germplasm can be explored further for rice breeding as reported previously (Lu 2013). Genetic diversity of the same W populations estimated in this study was much greater than that in previous studies based on SSR fingerprints (Yang *et al.* 2004; Li *et al.* 2006), probably due to different SSR loci applied and number of W samples included for analyses. In addition, the E-population showed a high level of observed heterozygosity in this study, confirming of its relatively frequent introgression with diverse rice cultivars through time. The high level of heterozygosity from crop-wild introgression may lead to the uncertainty of this population, particularly by the genetic swapping effect (Todesco *et al.* 2016).

Cultivated rice and its wild ancestor *O. rufipogon* can produce highly fertile hybrids from natural hybridization, owing to their close genetic relationship and high compatibility (Oka and Chang 1961). This wild ancestor has a relatively high outcrossing rate, ranging from ~5 to 60 % (Oka and Morishima 1967). Findings from previous reports and this study already indicated the considerable influences of crop-wild gene flow or introgression on genetic differentiation and structure of the wild *O. rufipogon* populations. In addition, such crop-wild introgression may lead to genetic swamping, meaning the replacement of pure parental genotypes of wild populations in crop-wild hybrids (Todesco *et al.* 2016), because crop-wild hybrids in rice could survive and produce highly fertile hybrids. Previous studies also showed that introgression from crop to wild relatives could result in genetic swamping of wild relatives, and this type of genetic swamping may lead to the local extinction of wild relatives (Hegde *et al.* 2006; Scarcelli *et al.* 2017). Similarly, genetic swamping in wild rice populations by introgression-resulted crop-wild hybrids may increase the risk of the local extinction of endangered *O. rufipogon* populations, posing a challenge to the conservation of endangered species that are genetically related and spatially close to domesticated species.

Gene flow or introgression, particularly transgene flow/introgression, from crops to their wild relative populations or species may result in a wide range of consequences as suggested by many researchers (Song *et al.* 2003a; Wang *et al.* 2006; Lu and Yang 2009; Ellstrand *et al.* 2013; Lu 2016). Crop genes that convey fitness advantages, such as resistance to biotic and abiotic stresses, may promote invasive weeds (Ellstrand and Elam 1993; Lu and Yang 2009; Ellstrand *et al.* 2013). On the other hand, crop genes that convey fitness disadvantages may cause the extinction of endangered CWR species (Ellstrand and Elam 1993; Lu and Yang 2009; Lu 2013). Ellstrand *et al.* (1999) reported the world’s 13 most important food crops, of which 12 can hybridize with their wild relatives. This report re-emphasized the concerns over crop-wild gene flow or introgression. One aspect for the concerns is that crop-wild (trans)gene introgression increased the uncertainty of CWR conservation, which may alter the genetic integrity and evolution destiny of CWR (Ellstrand and Elam 1993; Lu and Yang 2009; Lu 2013). Results from this study demonstrated allelic introgression of cultivated rice, which can affect the genetic differentiation, structure and integrity of *O. rufipogon* populations, probably without the involvement of a selectively advantageous (trans)genes. Given that transgenic rice may be eventually released for commercial production in China (Lu 2016), impact of transgene introgression from GE rice varieties on wild *Oryza* species should be assessed.

It is widely accepted that genetic resources harboured in CWR populations or species are extremely valuable for crop breeding. However, due to habitat changes and disturbances by human activities, effective *in situ* conservation of CWR genetic resources, particularly those in small populations, becomes very difficult (Ellstrand and Elam 1993; Lu 2013). The commonly adopted strategies or methods for *in situ* conservation are to interrupt disturbances to the conserved CWR populations/species. The studied case in Gaozhou exemplifies such conservation strategies in which the local government built up metal-wire fences to surround the W populations with the aims of ensuring no disturbances (including animal grazing) to the protected areas. However, genetic impact of natural introgression from their crops on the protected W populations was not taken into consideration for this type of *in situ* conservation, where rice cultivars are extensively planted near the conservation site. Results from our study clearly indicated that crop-wild introgression could considerably alter the genetic structure and relationships of the protected wild rice populations, particularly those occurring in the close vicinity of cultivated rice fields. In other words, extensive crop-wild introgression may significantly change the genetic integrity and structure of CWR populations, even though they are under *in situ* conservation. Therefore, questions concerning the long-term impact of crop-wild allelic introgression on genetic integrity/diversity and evolutionary destiny of CWR populations and species in agroecosystems should be further studied for the effective *in situ* conservation.

## Conclusions

Crop-wild gene flow and allelic introgression considerably alters the genetic structure, relationship and integrity of the wild rice (*O. rufipogon*) populations that are under *in situ* conservation, although such introgression seemed not to change their overall genetic diversity. Our findings have broad implications for the *in situ* conservation of endangered CWR species/populations, particularly for those that are genetically and spatially close to their domesticated counterparts. The *in situ* conservation strategy for CWR species/populations only by means of a physical isolation (e.g. metal-wire or other fences) to interrupt disturbances from outside sources may not be sufficient, particularly at the sites with extensive cultivation of their domesticated species. Probably a buffer zone with properly determined spatial distances or other types of cultivated crops can be established to limit crop-to-wild allelic introgression for maintaining the genetic integrity of the *in situ* protected W populations. In addition, restoration of ecological habitats that are suitable for CWR populations/species may also be useful for *in situ* conservation, as proposed by Todesco *et al.* (2016) that the mature, diverse and undisturbed habitats may become more resistant to hybrid establishment and genetic swamping.

## Supporting Information

The following additional information is available in the online version of this article—

**Table S1.** Name of the rice cultivars (CV-1) collected from southern China (including Guangdong, Guangxi and Fujian provinces) for SSR (simple sequence repeat) marker analysis.

**Table S2.** Name of the rice cultivars (CV-2) collected from Gaozhou and its neighbouring regions in Guangdong Province, China, for InDel (insertion/deletion) marker analysis.

**Table S3.** Name of the 34 SSR primer pairs and their location on chromosomes used in this study (http://www.gramene.org).

**Table S4.** Name of the 34 InDel primer pairs and their location on chromosomes used in this study from Lu *et al.* (2009).

**Table S5.** Frequencies of shared alleles by cultivated rice (*Oryza sativa*, CV-1) and wild rice (*O. rufipogon*) populations (A–F) at 16 SSR loci. ‘-’ indicates no relevant allele detected.

**Table S6.** Frequencies of shared alleles by the populations (A–F) of perennial common wild rice (*Oryza rufipogon*) and rice cultivars at different InDel loci. ‘-’ indicates no relevant allele detected.

**Table S7.** Genetic diversity parameters for wild rice (*Oryza rufipogon*) populations (A–F) in Gaozhou compared with those of cultivated rice controls (CV-2 and CV-1) from southern China based on InDel fingerprints.

## Sources of Funding

This research is supported by the Nature Science Foundation of China (31330014, 31271683), National Program of Development of Transgenic New Species of China (2016ZX08011-006) and ‘Gan-Po Talent 555’ Project of Jiangxi Province, China.

## Contributions by the Authors

B.-R.L., C.L. conceived and designed the experiments. X.J., Y.C., P.L., X.X.C., B.-R.L. performed the experiments. X.J., Y.C., B.-R.L., J.R., C.L. analysed the data. X.J., Y.C., J.R., B.-R.L. wrote the manuscript.

## Conflicts of Interest

None declared.

## Supplementary Material

Supplementary Materials
