# Supplementary material for: Introgression from cultivated rice alters genetic structures of wild relative populations: implications for in situ conservation
Source: AoB Plants. 2017 Oct 16;10(1):plx055. doi: 10.1093/aobpla/plx055 (PMC5751058; doi:10.1093/aobpla/plx055)
Supplement: Supplementary Materials [file plx055_suppl_supplementary_materials.docx]

**Table S1** Name of the rice cultivars (CV-1) collected from southern China (including Guangdong, Guangxi, and Fujian provinces) for SSR (simple sequence repeat) marker analysis

| No. | Name | Region | No. | Name | Region |
| --- | --- | --- | --- | --- | --- |
| 1 | Ai Jiao Nan Te | Guangdong | 19 | Qi Yue Xian | Guangxi |
| 2 | Guang Lu Ai 4 Hao | Guangdong | 20 | Liu Sha 1 Hao | Guangxi |
| 3 | Gui Chao 2 Hao | Guangdong | 21 | Guang Lu Ai15 | Guangxi |
| 4 | Huang Si Gui Zhan | Guangdong | 22 | Zao Shu Xiang Hei | Guangxi |
| 5 | Shu Ya Zhan | Guangdong | 23 | Me Mi | Guangxi |
| 6 | Si Miao | Guangdong | 24 | Hong Ai Nuo | Guangxi |
| 7 | E Si Niu | Guangdong | 25 | Ai Zi Zhan | Guangxi |
| 8 | Qi Mei | Guangdong | 26 | Hong Jing Han Gu | Guangxi |
| 9 | Nan Xiong Zao You | Guangdong | 27 | Heng Xian Liang Chun | Guangxi |
| 10 | Bai Ke Hua Luo | Guangdong | 28 | Jin You 1 Hao | Fujian |
| 11 | Hei Du 4 | Guangdong | 29 | Jin Bao Yin | Fujian |
| 12 | Chi Ke Nuo | Guangdong | 30 | Min Bei Wan Xian | Fujian |
| 13 | San Li Cun | Guangdong | 31 | Lu Cai Hao | Fujian |
| 14 | Bao Xuan 21 Hao | Guangdong | 32 | Yi Zhi Xiang | Fujian |
| 15 | Er Gang Ai | Guangdong | 33 | Hong Wan 1 Hao | Fujian |
| 16 | Xo Shi 15 | Guangdong | 34 | Yan Shui Chi | Fujian |
| 17 | Qing Si Ai 16B | Guangdong | 35 | Wu Ke Zhan | Fujian |
| 18 | Guang Ke Hei Nuo | Guangxi |  |  |  |

**Table S2** Name of the rice cultivars (CV-2) collected from Gaozhou and its neighboring regions in Guangdong province, China for InDel (insertion/deletion) marker analysis

| No. | Name | No. | Name | No. | Name | No. | Name |
| --- | --- | --- | --- | --- | --- | --- | --- |
| 1 | Da Nuo | 15 | Heng Ye | 29 | Yue Xiang Zhan | 43 | Long Xia Zhong |
| 2 | Bai Nuo Zi | 16 | Xian You 998 | 30 | Yue Xiu Zhan | 44 | Bei Yi Da Bai |
| 3 | Yu Tuan Nuo | 17 | You You 998 | 31 | Zhong Er Ruan Zhan | 45 | Tian Ji Bao |
| 4 | Da Nuo | 18 | Tian You 3550 | 32 | Zao Bai | 46 | Gao Jiao Zao Zi |
| 5 | Long Gou Wei | 19 | Qiu You 998 | 33 | Guang Xi Zhan | 47 | Wan Zhong |
| 6 | Yu Zhua | 20 | Tian You 368 | 34 | Zao Po Zi | 48 | Hei Jun Zi |
| 7 | Dun Di Lei | 21 | Bo You 998 | 35 | San Nuo Bai | 49 | Zao Nan Huang |
| 8 | San Gu | 22 | Yue Za 889 | 36 | Xia Zhi Bai | 50 | Zao Da Zhong |
| 9 | You Wei Zhan | 23 | Zhen You 998 | 37 | Bai Jing Zi |  |  |
| 10 | Tian Ji Du | 24 | Xian You 3550 | 38 | Huang Jin Zhan |  |  |
| 11 | Bai Yin 1 Hao | 25 | Tian You 122 | 39 | Huang Zhan |  |  |
| 12 | Wen Zi Zhan | 26 | Feng You 998 | 40 | Shen You Gao Zhan |  |  |
| 13 | Ken Si Ji | 27 | Jin Dao 138 | 41 | Zhu Zao |  |  |
| 14 | Hua Luo Zhan | 28 | Tian You 998 | 42 | Yin Ya Zhan |  |  |

**Table S3** Name of the thirty-four SSR primer pairs and their location on chromosomes used in this study (<http://www.gramene.org>)

| Primer | Chr. | Primer | Chr. | Primer | Chr. | Primer | Chr. |
| --- | --- | --- | --- | --- | --- | --- | --- |
| RM259 | 1 | RM307 | 4 | RM234 | 7 | RM467 | 10 |
| RM23 | 1 | RM471 | 4 | RM18 | 7 | RM258 | 10 |
| RM104 | 1 | RM348 | 4 | RM248 | 7 | RM228 | 10 |
| RM240 | 2 | RM161 | 5 | RM337 | 8 | RM202 | 11 |
| RM250 | 2 | RM13 | 5 | RM25 | 8 | RM224 | 11 |
| RM29 | 2 | RM598 | 5 | RM264 | 8 | RM247 | 12 |
| RM251 | 3 | RM217 | 6 | RM245 | 9 | RM235 | 12 |
| RM16 | 3 | RM50 | 6 | RM242 | 9 |  |  |
| RM130 | 3 | RM541 | 6 | RM205 | 9 |  |  |

**Table S4** Name of the thirty-four InDel primer pairs and their location on chromosomes used in this study from Lu *et al*. (2009)

| Primer | Chr. | Primer | Chr. | Primer | Chr. | Primer | Chr. |
| --- | --- | --- | --- | --- | --- | --- | --- |
| R1M7 | 1 | R3M23 | 3 | R6M44 | 6 | R10M30 | 10 |
| R1M30 | 1 | R3M30 | 3 | R7M7 | 7 | R10M40 | 10 |
| R1M37 | 1 | R3M53 | 3 | R7M37 | 7 | R11M23 | 11 |
| R1M47 | 1 | R4M13 | 4 | R8M23 | 8 | R11M40 | 11 |
| R2M10 | 2 | R4M17 | 4 | R8M33 | 8 | R12M10 | 12 |
| R2M24 | 2 | R4M43 | 4 | R8M46 | 8 | R12M27 | 12 |
| R2M26 | 2 | R5M13 | 5 | R9M10 | 9 | R12M43 | 12 |
| R2M50 | 2 | R5M30 | 5 | R9M42 | 9 |  |  |
| R3M10 | 3 | R6M14 | 6 | R10M17 | 10 |  |  |

Lu B-R, Cai X, Jin X. 2009. Efficient *indica* and *japonica* rice identification based on the InDel molecular method: its implication in rice breeding and evolutionary research. *Progress in Natural Science* **19**: 1241-1252.

**Table S5** Frequencies of shared alleles by cultivated rice (*Oryza sativa*, CV-1) and wild rice (*O. rufipogon*) populations (A~F) at 16 SSR loci. “-” indicates no relevant allele detected.

| SSR locus^1^ | ID of alleles | Frequency of the specific allele in CV-1 | Frequency of the specific allele in wild rice populations | | | | | |
| --- | --- | --- | --- | --- | --- | --- | --- | --- |
|  |  |  | A | B | C | D | E | F |
| RM130 | C | 0.91 | 0.03 | 0.07 | - | 0.35 | 0.50 | 0.02 |
| RM348 | C | 0.89 | 0.01 | 0.03 | - | - | 0.46 | 0.03 |
| RM234 | J | 0.70 | 0.05 | - | - | 0.22 | 0.44 | 0.01 |
| RM240 | I | 0.54 | - | 0.19 | - | - | - | 0.01 |
| RM25 | F | 0.46 | - | - | - | - | 0.44 | 0.11 |
| RM467 | E | 0.43 | 0.03 | - | - | - | 0.50 | 0.10 |
| RM337 | G | 0.34 | - | 0.02 | - | - | 0.02 | 0.01 |
| RM217 | P | 0.32 | - | - | - | - | 0.41 | 0.01 |
| RM50 | K | 0.31 | - | - | - | - | 0.46 | 0.02 |
| RM471 | G | 0.27 | - | - | - | 0.05 | 0.04 | - |
| RM337 | B | 0.24 | - | 0.06 | - | - | 0.46 | - |
| RM259 | E | 0.23 | 0.04 | 0.27 | - | 0.20 | 0.06 | - |
| RM248 | G | 0.21 | - | - | - | - | 0.48 | 0.01 |
| RM258 | F | 0.21 | - | 0.18 | - | - | - | - |
| RM50 | M | 0.20 | 0.01 | 0.29 | - | - | - | - |
| RM228 | Q | 0.15 | 0.02 | 0.01 | - | 0.33 | - | - |
|  | Average | **0.40** | **0.01** | **0.07** | **0.00** | **0.07** | **0.27** | **0.02** |

^1^ SSR loci that do not have a shared allele between cultivated and wild rice are not included.

**Table S6.** Frequencies of shared alleles by the populations (A~F) of perennial common wild rice (*Oryza rufipogon*) and rice cultivars at different InDel loci. “-” indicates no relevant allele detected.

| InDel locus | ID of alleles | Frequency of the specific allele in CV-2 | Frequency of the specific allele in wild rice populations (A~F) | | | | | |  |
| --- | --- | --- | --- | --- | --- | --- | --- | --- | --- |
|  |  |  | A | B | C | D | E | F | |
| R1M37 | A | 0.18 | 0.98 | 0.93 | 0.95 | 0.90 | 0.54 | 0.91 | |
|  | B | 0.82 | 0.02 | 0.07 | 0.05 | 0.10 | 0.46 | 0.09 | |
| R2M10 | A | 0.06 | 0.95 | 0.56 | 0.63 | 0.65 | 0.46 | 0.36 | |
|  | B | 0.94 | 0.05 | 0.44 | 0.37 | 0.35 | 0.54 | 0.64 | |
| R2M26 | A | 0.07 | 0.58 | 1.00 | 1.00 | 0.70 | 0.52 | 0.93 | |
|  | B | 0.93 | 0.42 | - | - | 0.30 | 0.48 | 0.07 | |
| R2M50 | A | 0.10 | 0.07 | 0.38 | 0.07 | 0.35 | 0.23 | 0.06 | |
|  | B | 0.90 | 0.93 | 0.63 | 0.93 | 0.65 | 0.77 | 0.94 | |
| R3M10 | A | 0.12 | 0.18 | 0.06 | 0.54 | 0.55 | 0.38 | 0.24 | |
|  | B | 0.88 | 0.82 | 0.94 | 0.46 | 0.45 | 0.63 | 0.76 | |
| R3M23 | A | 0.08 | 0.05 | 0.31 | 0.51 | 0.30 | 0.10 | 0.58 | |
|  | B | 0.92 | 0.95 | 0.69 | 0.49 | 0.70 | 0.90 | 0.42 | |
| R3M30 | A | 0.08 | 0.90 | 0.36 | 0.58 | 0.35 | 0.06 | 0.43 | |
|  | B | 0.92 | 0.10 | 0.64 | 0.42 | 0.65 | 0.94 | 0.57 | |
| R4M13 | A | 0.08 | 0.98 | 0.82 | 1.00 | 0.85 | 0.63 | 0.94 | |
|  | B | 0.92 | 0.02 | 0.18 | - | 0.15 | 0.38 | 0.06 | |
| R4M17 | A | 0.08 | 0.99 | 0.74 | 1.00 | 0.90 | 0.54 | 0.97 | |
|  | B | 0.92 | 0.01 | 0.26 | - | 0.10 | 0.46 | 0.03 | |
| R4M43 | A | 0.09 | 0.05 | 0.22 | 0.49 | 0.10 | 0.48 | 0.10 | |
|  | B | 0.91 | 0.95 | 0.78 | 0.51 | 0.90 | 0.52 | 0.90 | |
| R5M13 | A | 0.39 | 0.01 | 0.02 | 0.04 | - | - | 0.01 | |
|  | B | 0.61 | 0.99 | 0.98 | 0.96 | 1.00 | 1.00 | 0.99 | |
| R8M23 | B | 1.00 | 0.94 | 0.61 | 0.82 | 0.45 | 0.85 | 0.73 | |
| R9M10 | A | 0.47 | 1.00 | 0.97 | 1.00 | 1.00 | 1.00 | 0.83 | |
|  | B | 0.53 | - | 0.03 | - | - | - | 0.17 | |
| R9M42 | A | 0.14 | 0.97 | 0.80 | 0.43 | 0.55 | 0.48 | 0.84 | |
|  | B | 0.86 | 0.03 | 0.20 | 0.57 | 0.45 | 0.52 | 0.16 | |
| R10M17 | A | 0.10 | 0.94 | 0.83 | 0.93 | 0.75 | 0.54 | 0.92 | |
|  | B | 0.90 | 0.06 | 0.17 | 0.07 | 0.25 | 0.46 | 0.08 | |
| R11M23 | A | 0.09 | 0.86 | 0.70 | 0.99 | 0.30 | 0.94 | 0.86 | |
|  | B | 0.91 | 0.14 | 0.30 | 0.01 | 0.70 | 0.06 | 0.14 | |
| R12M10 | A | 0.09 | 0.01 | 0.02 | - | - | 0.02 | 0.12 | |
|  | B | 0.91 | 0.99 | 0.98 | 1.00 | 1.00 | 0.98 | 0.88 | |
| R12M27 | B | 1.00 | 0.04 | 0.06 | 0.55 | 0.25 | 0.88 | 0.14 | |
| R1M7 | A | 0.97 | - | 0.40 | - | 0.05 | 0.52 | 0.09 | |
|  | B | 0.03 | 1.00 | 0.60 | 1.00 | 0.95 | 0.48 | 0.91 | |
| R1M30 | A | 0.86 | 0.02 | 0.16 | 0.05 | 0.10 | 0.48 | 0.03 | |
|  | B | 0.14 | 0.98 | 0.84 | 0.95 | 0.90 | 0.52 | 0.97 | |
| R1M47 | A | 0.91 | 0.01 | 0.30 | 0.53 | 0.50 | 0.71 | 0.02 | |
|  | B | 0.09 | 0.99 | 0.70 | 0.47 | 0.50 | 0.29 | 0.98 | |
| R2M24 | A | 0.92 | 0.92 | 0.31 | 1.00 | 0.65 | 0.90 | 0.68 | |
|  | B | 0.08 | 0.08 | 0.69 | - | 0.35 | 0.10 | 0.32 | |
| R3M35 | A | 0.82 | 0.22 | 0.10 | - | 0.10 | 0.02 | 0.03 | |
|  | B | 0.18 | 0.78 | 0.90 | 1.00 | 0.90 | 0.98 | 0.97 | |
| R5M30 | A | 0.92 | 0.04 | 0.18 | - | 0.05 | 0.50 | 0.12 | |
|  | B | 0.08 | 0.96 | 0.82 | 1.00 | 0.95 | 0.50 | 0.88 | |
| R6M14 | A | 0.63 | 0.01 | 0.41 | - | 0.20 | 0.54 | 0.03 | |
|  | B | 0.37 | 0.99 | 0.59 | 1.00 | 0.80 | 0.46 | 0.97 | |
| R6M44 | A | 0.88 | 0.04 | 0.22 | 0.01 | - | 0.44 | 0.11 | |
|  | B | 0.12 | 0.96 | 0.78 | 0.99 | 1.00 | 0.56 | 0.89 | |
| R7M7 | A | 0.66 | 0.03 | 0.28 | - | 0.15 | 0.13 | 0.31 | |
|  | B | 0.34 | 0.97 | 0.72 | 1.00 | 0.85 | 0.88 | 0.69 | |
| R7M37 | A | 0.70 | 0.57 | 0.97 | 0.75 | 1.00 | 0.96 | 0.97 | |
|  | B | 0.30 | 0.43 | 0.03 | 0.25 | - | 0.04 | 0.03 | |
| R8M33 | A | 0.94 | 0.01 | 0.02 | 0.05 | 0.35 | 0.46 | 0.10 | |
|  | B | 0.06 | 0.99 | 0.98 | 0.95 | 0.65 | 0.54 | 0.90 | |
| R8M46 | A | 0.84 | 0.03 | 0.11 | 0.70 | 0.30 | 0.88 | 0.08 | |
|  | B | 0.16 | 0.97 | 0.89 | 0.30 | 0.70 | 0.13 | 0.92 | |
| R10M30 | A | 1.00 | 0.03 | 0.06 | 0.70 | 0.35 | 0.44 | 0.06 | |
| R10M40 | A | 0.86 | 0.08 | 0.05 | 0.72 | 0.30 | 0.46 | 0.09 | |
|  | B | 0.14 | 0.92 | 0.95 | 0.28 | 0.70 | 0.54 | 0.91 | |
| R11M40 | A | 0.76 | 0.03 | 0.06 | 0.05 | 0.05 | 0.02 | 0.03 | |
|  | B | 0.24 | 0.97 | 0.94 | 0.95 | 0.95 | 0.98 | 0.97 | |
| R12M43 | A | 0.82 | 0.09 | 0.23 | 0.04 | - | 0.44 | 0.23 | |
|  | B | 0.18 | 0.91 | 0.77 | 0.96 | 1.00 | 0.56 | 0.77 | |
|  | Average | **0.52** | **0.49** | **0.49** | **0.51** | **0.49** | **0.51** | **0.49** | |

**Table S7** Genetic diversity parameters for wild rice (*Oryza rufipogon*) populations (A~F) in Gaozhou compared with those of cultivated rice controls (CV-2 and CV-1) from southern China based on InDel fingerprints.

| Population code | *A^1^* | *A*_e_ | *H*_o_ | *H*_e_ | *I* | DG | Total no. of alleles | No. of alleles specific to rice cultivars or wild rice populations |
| --- | --- | --- | --- | --- | --- | --- | --- | --- |
| Wild rice -A | 1.941 | 1.160 | 0.112 | 0.115 | 0.208 | 0.116 | 66 | 0 |
|  | (0.041) | (0.040) | (0.034) | (0.021) | (0.030) | (0.022) |  |  |
| -B | 1.971 | 1.436 | 0.276 | 0.269 | 0.417 | 0.272 | 67 | 0 |
|  | (0.029) | (0.055) | (0.038) | (0.028) | (0.037) | (0.028) |  |  |
| -C | 1.676 | 1.348 | 0.204 | 0.197 | 0.298 | 0.200 | 57 | 0 |
|  | (0.081) | (0.070) | (0.054) | (0.036) | (0.049) | (0.036) |  |  |
| -D | 1.824 | 1.481 | 0.276 | 0.282 | 0.423 | 0.297 | 62 | 0 |
|  | (0.066) | (0.062) | (0.037) | (0.031) | (0.043) | (0.033) |  |  |
| -E | 1.941 | 1.615 | **0.482** | 0.334 | 0.490 | 0.341 | 66 | 0 |
|  | (0.041) | (0.070) | (0.067) | (0.033) | (0.042) | (0.033) |  |  |
| -F | 2.000 | 1.311 | 0.189 | 0.209 | 0.346 | 0.211 | 68 | 0 |
|  | (0.000) | (0.048) | (0.031) | (0.024) | (0.031) | (0.024) |  |  |
| Overall*^2^* | 1.892 | 1.392 | 0.257 | 0.234 | 0.364 | 0.239 | 68 | 0 |
|  | (0.022) | (0.026) | (0.020) | (0.013) | (0.017) | (0.013) |  |  |
| CV-2 | 1.912  (0.049) | 1.315  (0.045) | 0.123  (0.027) | 0.214  (0.023) | 0.353  (0.031) | 0.216  (0.023) | 65 | 0 |

*^1^ A*: number of alleles; *A*_e_: number of effective alleles; *H*_o_: observed heterozygosity; *H*_e_: expected heterozygosity; *I*: Shannon index; DG: Nei’s unbiased genetic diversity (Nei 1987). Numbers in parentheses indicate standard deviation.

*^2^* The overall values of genetic diversity parameters were calculated based all wild rice samples as a population.
